# Clarification? Yes! Standarization? No. Or: What Kind of Cooperation for the Sociology of Culture?

**DOI:** 10.1007/s12108-016-9309-x

**Published:** 2016-04-07

**Authors:** Monika Krause

**Affiliations:** Department of Sociology, Goldsmiths, University of London, 8 Lewisham Way, London, SE14 6NW UK

**Keywords:** Culture, Paradigms, Concepts, Definitions, American Sociology

## Abstract

Christian Smith’s paper “The Incoherence of ‘Culture’ in American Sociology” is a valuable provocation that can prompt us to reflect on the role of concepts and on the role of agreement on the definition of concepts in scientific research. In this comment paper, I raise questions about Smith’s empirical expectation that sociologists should agree on a concept of culture based on debates in the sociology of science. I also suggest that in terms of the future agenda for the sociology of culture, we should distinguish between dialogue and clarification on the one hand, which I agree is needed, and standardization on the other hand, which seems incompatible with open-minded empirical research. Rather than work on agreement on what culture is, we might work on clarifying relevant distinctions among dimensions of culture.

For his paper “The Incoherence of ‘Culture’ in American Sociology”, Christian Smith has surveyed a range of social scientific publications on culture in the US and notes that authors use a large and disparate set of concepts for culture. For Smith, this lack of agreement is a problem for the credibility and substance of the field, and he urges scholars to move towards a more coherent and standardized concept of culture.

Smith’s observations about the literature offer a valuable provocation, and in my response, I will not offer a strong alternative interpretation of his data. I do want to raise two sets of cautions and questions regarding Smith’s conclusions, on points where I think Smith’s writing gestures towards more clarity than the paper really offers; cautions and questions that we might want to consider as we address the larger questions that Smith’s paper itself is addressing. These larger questions concern on the one hand empirical questions about the role that concepts and in particular the role that agreement on the definitions of concepts play in scientific inquiry. They concern, on the other hand, a prescriptive discussion regarding how we should proceed with issues of conceptualization and research in the sociology of culture.

Regarding the first set of concerns, Smith, without quite saying so, compares the sociology of culture unfavorably to paradigmatic science. I want to question why we should expect work on culture to look like one paradigm, on either empirical, or prescriptive grounds. It is not clear that all successful research is organized around paradigms and it is not clear that even if we wanted sociology to be more strongly structured by paradigms, we would need the sociology of culture specifically to be organized around one paradigm.

Secondly, Smith’s paper relies on an opposition between coherence, standardization, agreement, clarity, discipline on the one hand, and incoherence, variety, disagreement, vagueness, contradiction and muddledness on the other hand, which obscures important choices and stakes that arise within each of these supposed alternatives. Smith raises useful questions for future work in the sociology of culture but I am not entirely sold on the package the paper seems to offer in terms of diagnosis or treatment. For what it is worth, I think, clarity and dialogue are a good thing, but standardization is not.

I am not convinced that we need to agree what culture “essentially is.” I am troubled by the limited role a proposal concerned with standardization and conceptual coherence seems to allocate to research. Rather than work on agreement on what culture is, we might work on clarifying relevant distinctions among dimensions of culture that could be investigated further.

Smith devotes a section of the paper, and some space in the conclusion, to preempting criticism of his expectation of coherence, but I think his discussion of these issues moves rather too quickly and I want to at least try to raise my cautions and questions about the paper without being put into either the “everything goes,” or the “chaos is great camp.” I too am interested in what (social) scientific practices can uniquely contribute in terms of observations about the world and how they can be improved. I think as a basis for answering these prescriptive questions textual analysis and the application of logic to texts has its limits, and we can benefit from closer observation of actual practices in social scientific work in the future.

## The Role of Agreement on Concepts in (Social) Scientific Research

How important, empirically, is conceptual coherence for scientific research? The classic answer to this is Thomas Kuhn’s. For Kuhn (1962), normal, mature science functions within paradigms; the definition of a “lexicon” of key terms, which do not overlap, is important to paradigms. He writes, “periods in which a speech community does deploy overlapping kind terms end in one of two ways: either one entirely displaces the other, or the community divides in two” (Kuhn 1993:319).

Smith, without really discussing Kuhn, seems to be shaped by this account and he compares cultural sociology unfavorably to it, both in empirical terms (“cultural sociology does not function like paradigmatic science”) and in prescriptive terms (“cultural sociology is deficient because it does not function like paradigmatic science”).

But I have questions both about Kuhn’s empirical account and about Smith’s prescriptive reading of it. First, it is not clear that research in all or many other fields of science is actually characterized by conceptual coherence or agreement on key terms. Second, it is not clear that conceptual agreement is important for the success of a field. Third, even if paradigmatic research has its role and unique advantages, it is not clear that we would expect it to emerge in any specific disciplinary subfield, or around any specific term across disciplines. That is, even if we think sociology as a discipline should look more like the paradigmatic science described by Kuhn, it is not clear that we should expect or want culture to be at the center of a paradigm.

Evelyn Fox-Keller has pointed out that Kuhn’s account, though often read as an analysis of “science in general,” is actually based on the case of theoretical physics. She suggests Kuhn’s account is an idealization even of theoretical physics, but that even if Kuhn was right about theoretical physics, we should not assume that other fields behave in a similar way (Fox-Keller 2012). Fox-Keller analyses contemporary biology and argues that there is no agreement as to what “environment,” “competition,” “inheritability,” or the “genome” means.

Biologists are rarely troubled by this polysemy, Fox-Keller notes, and by most accounts genetic biology is doing quite well as a field despite – or perhaps because of - this “incoherence.” It is worth emphasizing that it is not clear that all of this success is only “external” or “instrumental,” in terms of prestige, for example, or research funding. The ambiguity of key terms might help researchers in this field also in terms of more substantive criteria related to scientific progess such as creativity, a lack of dogmatism, and a focus on questions about the world (rather than about concepts).

Lastly, it is not clear, either empirically or prescriptively, how agreement on concepts relates to disciplinary or subdisciplinary boundaries. In other words, it is not clear that even if we wanted sociology to be more strongly structured by paradigms, we would expect or need the sociology of culture specifically to be organized around one paradigm.

Smith claims that “culture” is a term that is central to sociology, and he implies that a coherent concept of culture would thus be central to sociology’s credibility. But however much we sociologists of culture might like the term to be central to sociology, historically it seems to me it has rather been a residual category.

As a collection of people, as well, the culture section of the American Sociological Association seems to me for better or worse a “residual section,” partly a home for people with no other home, or rather a place where people who do have other homes can talk to each other about cross-cutting issues - Michele Lamont (2004) has described a similar pattern for the theory section. That makes it rather less surprising that a sample of texts partly based on section awards will not necessarily agree on key terms; and it does not necessarily follow that the section is not contributing to meaningful intellectual and scientific aims.

## The Way Forward for the Sociology of Culture

The real purpose of Smith’s diagnosis is not to discuss the role of agreement on concepts empirically but to provide a basis for prescription for the sociology of culture. Here, Smith’s paper relies on an opposition between coherence, standardization, agreement, clarity, discipline on the one hand and incoherence, variety, disagreement, muddledness, and relativism on the other hand.

This means that the argument proceeds as though cooperation, dialogue, clarity, agreement, coherence and standardization are all the same thing, which seems to me to obscure important subtleties in the choices we actually face as we proceed to work on and around culture, individually and collectively. There are certainly some things that Smith calls out as problematic, which I agree are bad. And there are some things on Smith’s list of positive terms that I would agree with are good. But that does not mean, we have to buy the packaging Smith proposes. Roughly speaking, I am for clarity, dialogue and cooperation, and I am against standardization.

Continued dialogue on conceptual matters is important. Smith’s charge that there is too little of it is worth taking seriously, as is his explanation as to why there is too little: Smith hints at the fact that scholars might avoid “engaging in sustained intellectual criticisms of and arguments with culture-friendly colleagues with whom they disagree about culture, yet who also share deeper oppositions to theory rivals … Better to live and let live” (Smith nd, p. 4). Smith might be right about this, and this would be a problematic tendency – though we may note that this is a tendency we can observe in many fields – partly fostered by some of the dysfunctional side-effects of peer-review: There is little incentive to criticise likely reviewers on questions of definition.

Smith wants more than dialogue and clarification, though. While he says he does not want to impose uniformity, he does advocate for standardization: Smith argues that “different culture theorists in the last few decades define culture as very many different things … culture simply cannot be all of these things … as a matter of ontological reality, most of the ideas and claims above are incommensurate or incompatible”. A practice, for instance, is a fundamentally different kind of thing from a cognitive representation, mental schema, idea or belief” (Smith 2016 page 14).

Rather than “sustained intellectual criticisms” and “arguments”, Smith here seems to advocate agreement and standardisation. Agreement can be on several levels; I do not see how agreement about what culture “essentially is” can be helpful for a progressive research program. Though Smith defends a rather strong notion of “external reality” and puts up relativism as one of the foes in the set-up of his argument, his scheme does not seem to leave a lot of space for empirical research and what it might discover about culture.

Surely in response to the analysis of practices, schemas and ideas, which we have accumulated, we want more discussion and cooperation around relevant distinctions that sensitize us to different aspects of culture rather than agreement on one concept of culture. Smith’s review highlights some of the distinctions, which we could discuss, and we could add others, such as the distinction between patterns of meaning-making that are shared – in agreement or disagreement on a societal level and meaningful practices specific to specific fields of practice, or the distinction between patterns of meaning-making that are specific to a specific time-period and those that are specific to a specific group. Though different accounts of culture can stress one aspect of culture over others, it makes no sense to discuss a priori, which of these is the “wrong definition”. Rather, we should ask through research, which dimension matters how, if at all, in particular empirical settings, and how they relate to each other.
